# DA-6 promotes germination and seedling establishment from aged soybean seeds by mediating fatty acid metabolism and glycometabolism

**DOI:** 10.1093/jxb/ery247

**Published:** 2018-07-03

**Authors:** Wenguan Zhou, Feng Chen, Sihua Zhao, Caiqiong Yang, Yongjie Meng, Haiwei Shuai, Xiaofeng Luo, Yujia Dai, Han Yin, Junbo Du, Jiang Liu, Gaoqiong Fan, Weiguo Liu, Wenyu Yang, Kai Shu

**Affiliations:** Key Laboratory of Crop Ecophysiology and Farming System in Southwest China, Institute of Ecological Agriculture, Sichuan Agricultural University, Chengdu, China

**Keywords:** Aged soybean seed, DA-6, fatty acid, germination, glycometabolism, seedling establishment

## Abstract

Soybean seeds contain higher concentrations of oil (triacylglycerol) and fatty acids than do cereal crop seeds, and the oxidation of these biomolecules during seed storage significantly shortens seed longevity and decreases germination ability. Here, we report that diethyl aminoethyl hexanoate (DA-6), a plant growth regulator, increases germination and seedling establishment from aged soybean seeds by increasing fatty acid metabolism and glycometabolism. Phenotypic analysis showed that DA-6 treatment markedly promoted germination and seedling establishment from naturally and artificially aged soybean seeds. Further analysis revealed that DA-6 increased the concentrations of soluble sugars during imbibition of aged soybean seeds. Consistently, the concentrations of several different fatty acids in DA-6-treated aged seeds were higher than those in untreated aged seeds. Subsequently, quantitative PCR analysis indicated that DA-6 induced the transcription of several key genes involved in the hydrolysis of triacylglycerol to sugars in aged soybean seeds. Furthermore, the activity of invertase in aged seeds, which catalyzes the hydrolysis of sucrose to form fructose and glucose, increased following DA-6 treatment. Taken together, DA-6 promotes germination and seedling establishment from aged soybean seeds by enhancing the hydrolysis of triacylglycerol and the conversion of fatty acids to sugars.

## Teaser

The plant growth regulator diethyl aminoethyl hexanoate (DA-6) promotes the germination and seedling establishment of aged soybean seeds through enhancing the conversion from triacylglycerol to fatty acids and sugars.

## Introduction

The legume species soybean (*Glycine max* L.) originated in East Asia (Kuroda *et al.*, 2006; Lam *et al.*, 2010), and is now widely grown, being the primary oilseed crop in the world, with the USA, Brazil, Argentina, India, and China being the main soybean-growing countries (Zhou *et al.*, 2015; Schulte *et al.*, 2017). In China, where soybean production depends on maize–soybean intercropping, the area under which soybean is grown has increased significantly in recent years (Yang *et al.*, 2014, 2015; Chen *et al.*, 2017; Liu *et al.*, 2017). However, it is noteworthy that, despite this, China is currently the major soybean-importing country globally; to meet the increasing demand for plant protein, oil, and food, further improvements in soybean production are essential.

Seed germination is one of the most important stages of the plant life cycle, contributing to the distribution of wild species, and to increased yield and quality of cultivated crop plants (Shu *et al.*, 2013, 2015, 2016*b*, *c*; Kan *et al.*, 2016; Rubio de Casas *et al.*, 2017). Generally, the emergence of the radicle indicates the completion of seed germination (Bewley, 1997; Shu *et al.*, 2016*c*). Immediately following seed germination, seedling establishment is another key developmental stage, whereby the seedling transitions from the heterotrophic to the autotrophic state (Eastmond, 2006; Quettier *et al.*, 2008; Chen and Thelen, 2010; Theodoulou and Eastmond, 2012; Eastmond *et al.*, 2015). Consequently, both seed germination and seedling establishment are essential for subsequent plant development. It is worth noting that both processes are powered by the energy that is stored in the seed itself (Eastmond, 2004, 2006; Quettier *et al.*, 2008; Chen and Thelen, 2010).

The mitochondrial FAD-dependent glycerol-3-P dehydrogenase:ubiquinone oxidoreductase (FAD-GPDH) pathway has been proposed to be involved in the breakdown of fatty acids and glycerol in plant seeds (Huang, 1975). Several elegant studies demonstrated that the FAD-GPDH cascade is particularly important in oilseed plants, by which the hydrolysis of triacylglycerol releases free fatty acids and glycerol, with the fatty acids and glycerol then being converted to sugars, which support the seed germination and seedling establishment processes (Eastmond, 2004, 2006; Quettier *et al.*, 2008; Theodoulou and Eastmond, 2012). The Arabidopsis *SDP6* (*Sugar-Dependent 6*) gene encodes FAD-dependent glycerol-3-P dehydrogenase (FAD-G3P), and, although *sdp6* mutant seeds are able to germinate, the seedlings exhibit a marked arrested growth phenotype in the absence of exogenous sucrose during the seedling establishment stage (Quettier *et al.*, 2008). This means that the transition from glycerol and fatty acids to sucrose is significantly impaired in *sdp6* seeds, with the exogenous supply of sucrose fully rescuing the arrested growth phenotype of the *sdp6* mutant (Quettier *et al.*, 2008).

Another key gene, *SDP1* (*Sugar-Dependent 1*), encoding a patatin-like domain-containing triacylglycerol lipase, also plays a key role during post-germinative growth (Eastmond, 2006). In *sdp1*, the hydrolysis of triacylglycerol is blocked, resulting in *sdp1* seeds also exhibiting an arrested growth phenotype in the absence of sucrose, mimicking the *sdp6* phenotype (Eastmond, 2006). Altogether, both the hydrolysis of triacylglycerol and the conversion of fatty acids and glycerol to sugars are important for the successful powering of seed germination and seedling establishment, with the sugar supply allowing the young seedlings to achieve the photosynthetic autotrophic state (Graham, 2008; Kelly *et al.*, 2011).

Numerous studies have demonstrated that, compared with the cereal crop seeds, including rice, wheat, and maize, soybean seeds contain much higher oil and fatty acid contents (Schmidt *et al.*, 2011; Lestari *et al.*, 2013; Dhakal *et al.*, 2014; Liu *et al.*, 2014; Li *et al.*, 2017; Teng *et al.*, 2017). During storage, seed respiration, a catabolic reaction, utilizes glucose and other biomolecules (principally oils and fatty acids), and, as a consequence, significantly shortens seed longevity and decreases the rates of seed germination and seedling establishment, even causing soybean seeds which had been stored for long periods to be incapable of germination (Barros *et al.*, 2017; Munz *et al.*, 2017). The ability of aged soybean seed to germinate decreases markedly as storage time increases; interestingly, the loss of germination potential also correlates with a decline in RNA integrity in soybean seeds following prolonged storage (Fleming *et al.*, 2017). Another study demonstrated that, during natural aging processes, phospholipase Dα (PLDα) affected the soybean seed phospholipid and triacylglycerol profiles, suggesting that suppression of PLDα activity in soybean seed has the potential to improve seed quality during long-term storage (Lee *et al.*, 2012). A decline in the germination ability of aged soybean seed significantly constrains soybean production, as it results in poor germination and seedling emergence from farm-saved seeds in the field. Therefore, it would be worthwhile developing an efficient method to promote the germination and seedling establishment of aged soybean seeds. Moreover, dissection of the precise physiological and molecular mechanisms underlying the reduced germination and seedling emergence capabilities of aged seeds will help us to better understand the soybean seed germination and seedling establishment processes.

Diethyl aminoethyl hexanoate (DA-6) is a novel artificial plant growth regulator, which can increase leaf chlorophyll content, and increase the photosynthetic rate and the rates of carbon and oxygen metabolism in plants (Yokoyama *et al.*, 1982; Zhang *et al.*, 2008; Jiang *et al.*, 2012). In agriculture, DA-6 has been registered for use on a range of crops, including cabbage, pakchoi, cotton, tomato, soybean, peanut, and maize (Jiang *et al.*, 2012). Furthermore, DA-6 also increased microalgal growth and simultaneously improved the quality and quantity of microalgal lipid for biodiesel production (Salama *et al.*, 2014; Jiang *et al.*, 2015), while treatment with DA-6, in combination with EDTA, appeared to be optimal for the remediation efficiency of *Lolium perenne* L. (perennial ryegrass) on lead-contaminated soil (He *et al.*, 2013). However, to date, minimal information is available on the role of DA-6 in seed science research especially on seed germination and early seedling establishment from aged soybean seeds.

Here, we report that DA-6 promotes germination and seedling establishment in naturally and artificially aged soybean seeds by increasing triacylglycerol hydrolysis, fatty acid metabolism, and glycometabolism. Several types of physiological and biochemical analyses and quantitative PCR (qPCR) assays demonstrated that DA-6 increased the hydrolysis of triacylglycerol and the conversion of fatty acids to sugars during imbibition of aged soybean seeds, and, consequently, increased seed germination and seedling establishment from aged soybean seeds. We believe that this effective treatment will significantly expand the potential applications of DA-6 in agricultural systems, especially in countries where farm-saved seed is at risk of deterioration during storage.

## Materials and methods

### Plant materials and growth condition

The prevailing soybean cultivar in Southwestern China, Nandou-12 (ND-12), was employed in this study. The seeds were grown in the modern agricultural research and development base of Sichuan Agricultural University (Chengdu, China), and were harvested at the same time. The elite soybean seeds were used for dry storage for different periods as described in the Results section. All the soybean seeds in our lab are stored in a closed container box at room temperature, and the humidity is <5%. Silicon dioxide was added to the box to maintain the dry conditions.

### Controlled deteriorate treatment assay

The assay of controlled deterioration treatment (CDT) was performed according to the protocol described elsewhere, with modifications (Chen *et al.*, 2012, 2016). Those studies investigated the seed longevity in Arabidopsis (Chen *et al.*, 2012, 2016), while this study focused on soybean seeds, thus some procedures are modified. Briefly, the soybean seeds were put into warm water (58 °C) for 20 min, and then the treated seeds were dried for 2 d at room temperature. Finally, the dried seeds were chosen for further germination analyses.

### Seed germination and seedling establishment

Soybean seeds were incubated in 9 cm Petri dishes on two layers of medium-speed qualitative filter paper. Twenty-five seeds were placed in each Petri dish and 20 ml of sterile water or 200 μM DA-6 solution was added. The dishes were incubated in a box at 25 °C (Sanyo Versatile Environmental Test Chamber MLR-350H, made in Japan) under dark conditions.

The germination experiments were performed at 25 °C and 60% relative humidity under dark conditions; the germination rates under dark conditions were recorded using a green safety light, according to a previous assay (Barrero *et al.*, 2014). Radicle emergence was scored at the indicated time points. For each germination test, ≥75 seeds per type of soybean seed were used, and three experimental replications were performed. Post-germination growth data including radicle length and fresh weight of germinated seeds were quantified 2–3 d after imbibition according to the particular experiment. For each germination test, the average germination percentage ±SE of experiments was calculated.

All the germinated and non-germinated soybean seeds (25 seeds per Petri dish) were transferred into soil and grown in greenhouses under 25 °C with 16 h light and 8 h dark conditions. Subsequently, after 2 weeks, the rates of seedling establishment, plant height, dry weight of seedlings, and total chlorophyll content were quantified according to the requirements of the experiments.

### Gene expression analysis

Total RNA preparation, ﬁrst-strand cDNA synthesis, and the qPCR assay were performed as in our previously described protocol (Shu *et al.*, 2016*a*). In detail, DNase I-treated total RNA (2 μg) was denatured and then subjected to reverse transcription using Moloney murine leukemia virus reverse transcriptase (200 U per reaction; Promega Corporation). Gene expression was quantiﬁed in the logarithmic phase using the expression of the housekeeping *GmTubulin* RNA as an internal control. Three biological replicates were performed for each experiment. Quantitative PCR was performed on a QuantStudio 6 Flex Real-Time PCR System (Thermo Fisher Scientific, USA), with a real-time detection system according to the manufacturer’s instructions with Vazyme™ AceQ qPCR SYBR Green Master mix, and data were calculated using the comparative C_T_ method (Schmittgen and Livak, 2008). Primer sequence for qPCR are shown in Supplementary Table 1 at *JXB* online.

### Quantification of total chlorophyll

About 1 cm^2^ of leaves (avoiding the thicker veins) were sampled with a puncher, and then were cut into filaments of ~5 mm in length and ~1 mm in width for further analysis. Subsequently, the filaments were placed in a graduated tube containing 5 ml of of 80% acetone, and the tubes were placed under dark conditions until the filaments had turned completely white (overnight). To compensate for the possible losses due to volatilization, the extraction can be made up to 5 ml with 80% acetone, and the solution in the tube was gently poured into the cuvette, and finally the level of total chlorophyll was analyzed according to the protocol described elsewhere (Xie *et al.*, 2007). The SpectraMax i3x Multi-Mode microplate reader (Molecular Devices, LLC, USA) was employed.

### Quantification of various sugars

Samples were taken at different time points (0, 12, 24, 36, and 48 h) during imbibition. After 15 min at 105 °C in an oven, they were dried at 75 °C until a constant weight was detected and were then ground in a clean mortar and put into an Eppendorf tube. A 50 mg aliquot of sample was put into a 10 ml graduated centrifuge tube with addition of 3 ml of 80% ethanol, and placed in a 80 °C water bath with constant stirring for 40 min. Next, the supernatant was collected after centrifugation at 5000 *g* for 10 min. The extracted solution was diluted to 50 ml with 80% ethanol after extracting three times for the sugar content analysis.

Sucrose content was measured by using the resorcinol method and estimated on the basis of the absorbance at a wavelength of 480 nm (Shi *et al.*, 2016). Fructose content was quantified according to the method published elsewhere (Cai *et al.*, 2016). Total soluble sugar analysis was performed by using the anthrone sulfuric acid method (Lei *et al.*, 2014). The SpectraMax i3x Multi-Mode microplate reader (Molecular Devices, LLC, USA) was employed.

### Fatty acid extraction and measurements

Soybean seeds were ground with liquid nitrogen and quantified by using a freeze drying system. Fatty acids were extracted from soybean seed powder according to our previously published protocol (Yang *et al.*, 2017). Briefly, 2 ml of *n*-hexane was added to the ground soybean seeds (50 mg per tube), followed by 15 min ultrasonic extraction (40 kHz), and then the samples were kept at room temperature for 3 h. Subsequently, the solution was centrifuged at 10000 rpm and 4 °C for 10 min. Next, the supernatant was mixed and 3 ml of 0.4 M methanolic potassium hydroxide solution (Me-OH) was added, with vortex oscillation for 30 s, and then kept at room temperature for 1 h. Next, we transferred the upper liquid layer to a 5 ml capacity bottle and added *n*-hexane up to 5 ml, and further injected the extract into the GC-MS system through a 0.45 μm organic phase filter.

A total of 37 fatty acid methyl ester (FAME) standard mixtures including common fatty acids (C4–C24) were purchased from Nu-chek-prep Inc. (USA). Identification and quantification of each fatty acid were carried using the methods described previously (Yang *et al.*, 2017). Three biological replications were performed. To explore the relationship among the contents of fatty acids in different samples, a heat map was created by the Illustrator software.

### Measurement of soluble invertase activity

A 100 mg aliquot of soybean seeds was ground in a chilled mortar with 8 ml of ice-cold sterile water, and subsequently it was transferred into a 10 ml bottle. Next, it was put into a refrigerator at 4 °C for 3 h, and then the solution was centrifuged at 4000 rpm and 4 °C for 10 min. Next, the standard curve determination and quantification of soluble invertase activity in different types of soybean seeds under imbibition were measured according to a previously published procedure (Tang *et al.*, 1996). Three biological replications were performed.

### Statistical analysis

The data, including germination rates, fresh weight, and radicle length of germinated seeds, and fatty acid and sugar quantification results, were analyzed using Student’s *t*-test (SPSS 19.0). Image J software was used to measure the length of radicles.

## Results

### Natural aging significantly decreases soybean seed germination and seedling establishment

**Fig. 1.**
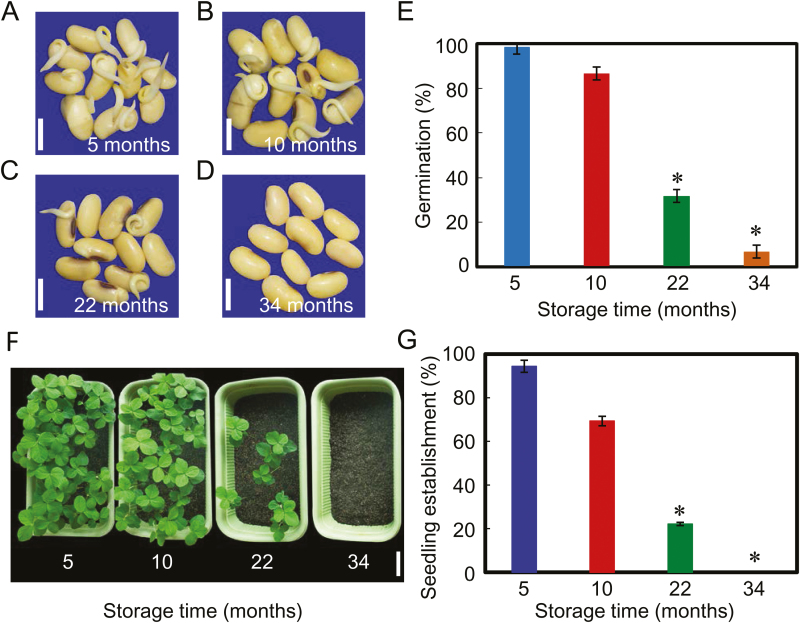
Natural aging significantly decreases soybean seed germination and seedling establishment abilities. (A–D) Representative photographs of naturally aged soybean seeds during the imbibition process (60 h after sowing). Soybean seeds were stored for 5, 10, 22, or 34 months after harvest and then subjected to analysis. Scale bar=10 mm. (E) The quantitative analysis of final germination rates of different samples (A–D) are shown (72 h after sowing). (F) The early seedling establishment phenotype of different samples (A–D) immediately following germination (2 weeks after sowing). Scale bar=100 mm. (G) The quantitative analysis of the seedling establishment rates of different samples are shown (2 weeks after sowing). Percentages are the average of three repeats ±SE. The germination experiments were performed under 25 °C and 60% relative humidity conditions, while the seedlings were grown under 25 °C and 16 h light with 8 h dark conditions. The asterisk (*) indicates a significant difference at *P*<0.0 by Student’s *t*-test analysis.

Soybean seed germination and seedling establishment were assessed in seeds subjected to different periods (i.e. 5, 10, 22, and 34 months) of dry storage. Seed germination analysis clearly showed that the germination rates of soybean seeds subjected to short-term storage (5 and 10 months) were significantly higher than those under long-term storage treatments (22 and 34 months) (Fig. 1A–E). The radicle length and fresh weight data of germinated seeds also supported the germination findings (Supplementary Fig. S1A, B). The final germination rate was nearly 100% and 85% for seeds stored for 5 and 10 months, respectively; however, the final germination rate of seeds stored for 22 months was only 30% (Fig. 1A–E), while almost all of the soybean seeds which had been stored for 34 months failed to germinate (Fig. 1D, E).

Next, we investigated the effect of soybean seed storage time on the early seedling establishment process. The same number of soybean seeds which were subjected to each of several different periods of storage were sown in soil, and then the percentage establishment of seedlings was scored. The results revealed that the seedling establishment rates from soybean seeds stored for short periods (5 or 10 months) were significantly higher than those from seeds stored for long periods (22 or 34 months) (Fig. 1F, G). Furthermore, it was noted that the seedling establishment rate after 14 d from seeds stored for 10 months (nearly 70%) was lower than their final germination rate (nearly 85%), and that a similar trend was also detected for seeds stored for 22 months (Fig. 1E, G). In particular, there was no seedling emergence from the seeds stored for 34 months, although their germination rate was ~10% (Fig. 1E, G). Taken together, these analyses confirmed the previous conclusion that the natural aging process significantly decreased the ability of soybean seeds to germinate and for seedlings to establish.

### DA-6 increases germination and seedling establishment from aged soybean seeds but not from fresh seeds

**Fig. 2.**
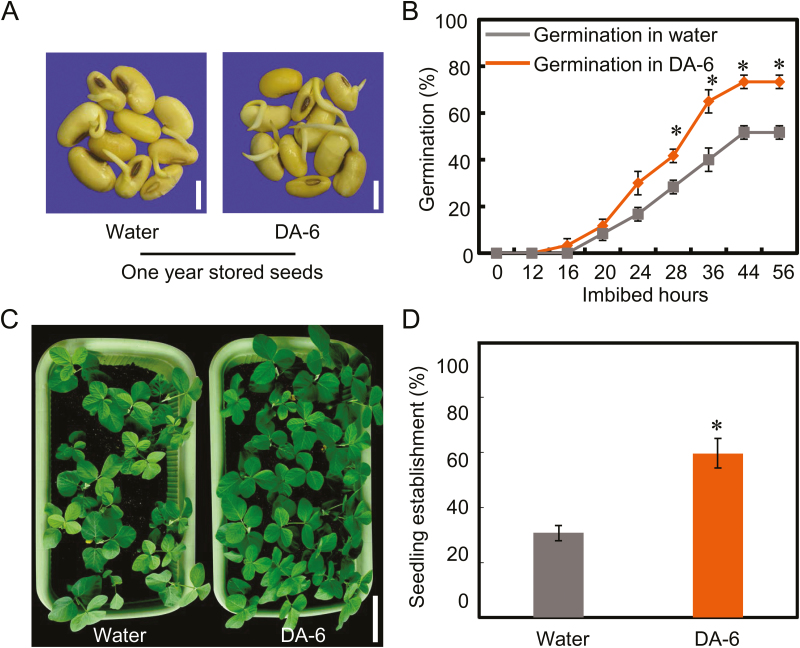
DA-6 promotes germination and seedling establishment from naturally aged soybean seeds. (A) Representative photographs of naturally aged soybean seeds (stored for 12 months) during the imbibition process (60 h after sowing), with or without DA-6 treatment. Scale bar=10 mm. (B) The quantitative analysis of final germination rates of seeds stored for 12 months in the absence or presence of DA-6 treatment. (C) The early seedling establishment phenotype of seeds stored for 12 months with or without DA-6 application. Scale bar=100 mm. (D) The statistical data of early seedling establishment for (C) are shown (15 d after sowing). Percentages are the average of three repeats ±SE. The germination experiments were performed under 25 °C and 60% relative humidity conditions, while the seedlings were grown under 25 °C and 16 h light with 8 h dark conditions. The asterisk (*) indicates a significant difference at *P*<0.05 by Student’s *t*-test analysis. A 200 μM concentration of exogenous DA-6 was used.

We had previously screened several plant growth regulators from those frequently employed in agriculture for their ability to promote germination and seedling establishment from aged soybean seeds (data not shown). Under our experimental conditions, we found that DA-6 increased germination and seedling establishment for both naturally and artificially aged soybean seeds. The results showed that DA-6 significantly increased the germination of naturally aged soybean seeds (stored for 12 months) (Fig. 2A, B). DA-6-treated naturally aged soybean seeds germinated more quickly than did the aged seeds without DA-6 treatment (Fig. 2A, B), and the data on the effects of DA-6 on radicle lengths and the fresh weights of germinated seedlings were consistent with the effects on germination (Supplementary Fig. S2A, B). Furthermore, DA-6 also promoted seedling establishment in naturally aged soybean seeds (Fig. 2C, D), with the seedling establishment rate of DA-6-treated aged seeds (60%) being twice that of seeds without DA-6 treatment (30%) (Fig. 2D).

Given that DA-6 increased germination of naturally aged soybean seeds (Fig. 2), we then investigated the effects of DA-6 on the germination of fresh soybean seeds. Soybean seeds which had been stored for 1 month (‘fresh seeds’) were employed in this experiment. The results showed that no effect of DA-6 on the germination or seedling establishment from fresh soybean seeds was detected (Supplementary Fig. S3A–D). These findings demonstrated that DA-6 promotes the germination of only aged soybean seeds, but not fresh seeds (Fig. 2; Supplementary Fig. S3).

**Fig. 3.**
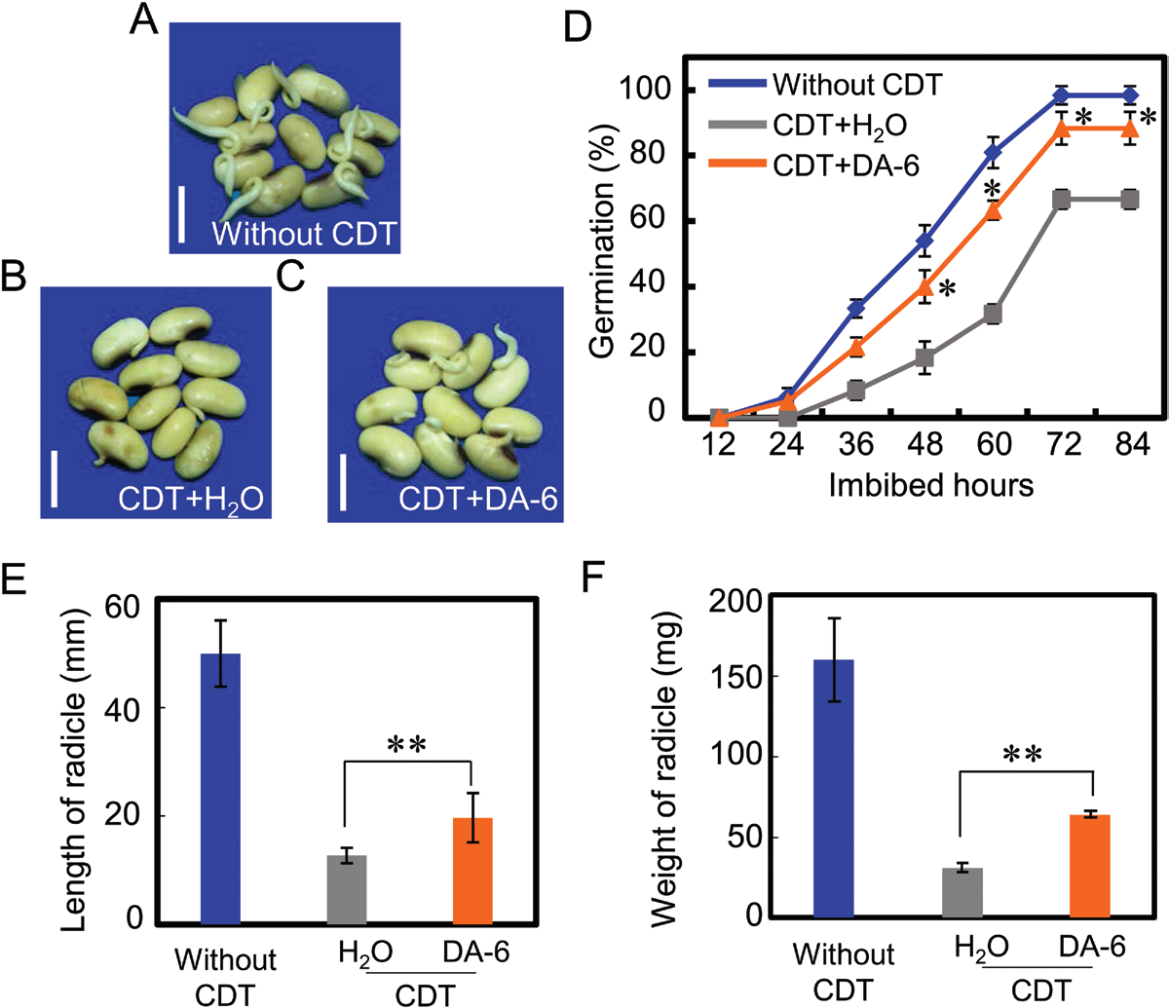
DA-6 enhances the germination ability of artificially aged soybean seeds. (A–C) Representative photographs of different types of soybean seeds (healthy seeds without CDT, CDT seeds with H_2_O, and CDT seeds with DA-6 treatment) during imbibition (48 h after sowing). Scale bar=10 mm. (D) The quantitative analysis of final germination rates of different samples (A–C) is shown. (E and F) The radical length (E) and fresh weight (F) of germinated soybean seeds were analyzed (48 h after sowing). The germination experiments were performed under 25 °C and 60% relative humidity with dark conditions; the germination rates under dark conditions were recorded using a green safety light, according to a previous assay (Barrero *et al.*, 2014). The average percentages of three repeats ±SE are shown. The asterisk (*) indicates a significant difference at *P*<0.05 by Student’s *t*-test analysis. A 200 μM concentration of exogenous DA-6 was used.

In order to control precisely the experimental conditions, we employed the CDT method, which is the prevailing artificial aging method used in seed longevity research (Bhattacharyya *et al.*, 1985; Das and Sen-Mandi, 1992; Chen *et al.*, 2012, 2016). Similar to the effect of DA-6 on germination and early seedling establishment from naturally aged soybean seeds (Fig. 2; Supplementary Fig. S2), the results showed that DA-6 also increased germination in artificially aged soybean seeds (Fig. 3A–D), while the radicle lengths and fresh weights of germinated seeds were also in line with the germination findings (Fig. 3E, F).

**Fig. 4.**
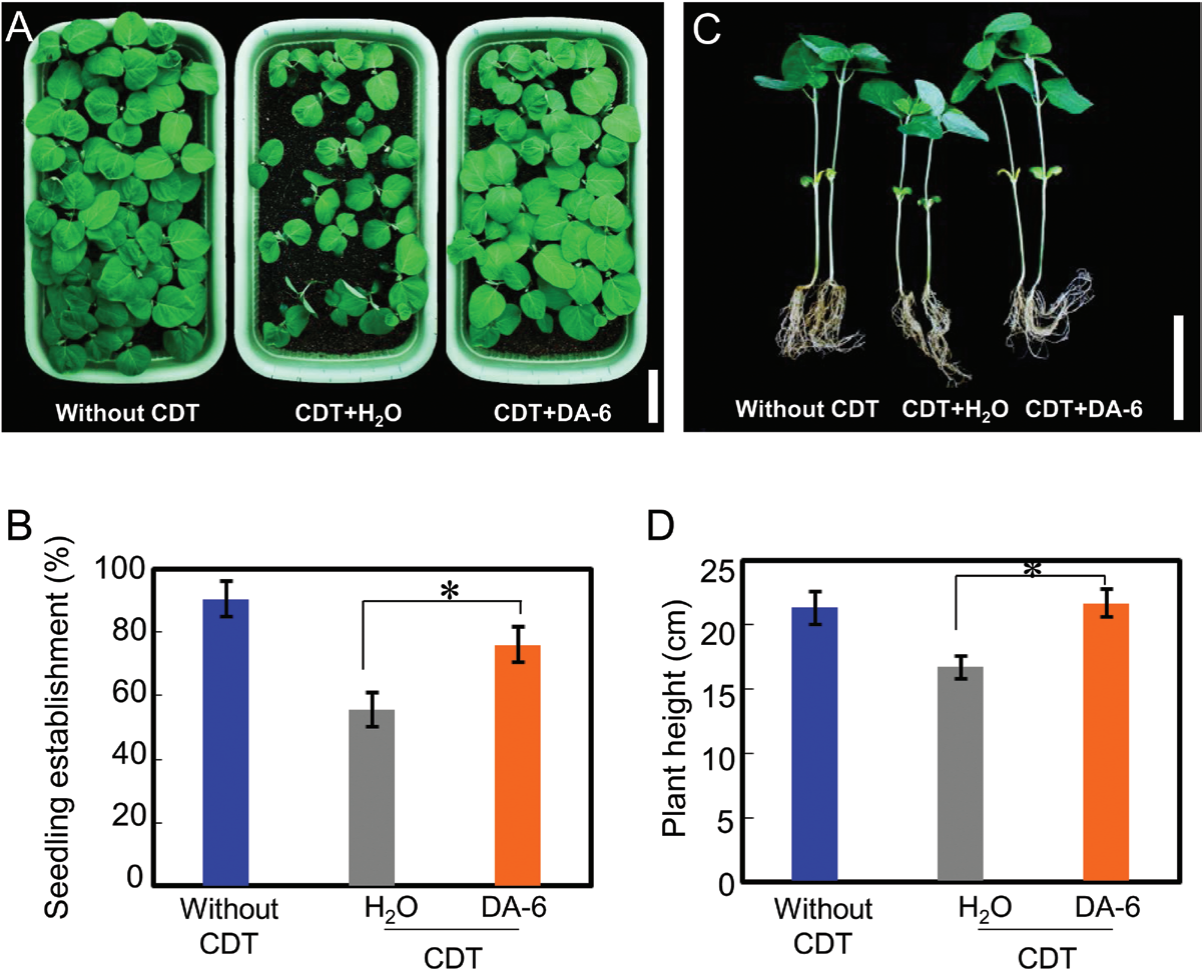
DA-6 positively regulates the seedling establishment of artificially aged soybean seeds. (A) The early seedling establishment phenotype of different types of soybean seeds (healthy seeds without CDT, CDT seeds with H_2_O, and CDT seeds with DA-6 treatment). Scale bar=100 mm. (B) The statistical data of early seedling establishment for (A) are shown (15 d after sowing). (C) Representative images of the height of soybean seedlings. Scale bar=100 mm. (D) The quantitative analysis of plant height for (C). Percentages are the average of three repeats ±SE. The seedlings were grown under 25 °C and 16 h light with 8 h dark conditions. The asterisk (*) indicates a significant difference at *P*<0.05 by Student’s *t*-test analysis. A 200 μM concentration of exogenous DA-6 was employed.

A positive effect of DA-6 on seedling establishment from artificially aged soybean seeds was also detected (Fig. 4A, B). The germination and seedling establishment rates of CDT-aged soybean seeds were lower than those from seeds without CDT, in both the presence and absence of DA-6 (Figs 3, 4). DA-6 also promoted the growth of seedlings from artificially aged soybean seeds, as illustrated by the corresponding values for the heights and dry weights of seedlings from aged seeds in the presence or absence of DA-6 (Fig. 4C, D; Supplementary Fig. S4A). DA-6 treatment also increased the total chlorophyll concentration in seedling true leaves (Supplementary Fig. S4B), a result which was consistent with findings from previous studies (Jiang *et al.*, 2012; Jiang *et al.*, 2015). Furthermore, given the finding that DA-6 resulted in similar positive effects on germination and seedling establishment from both naturally and artificially aged soybean seeds, subsequent experiments employed only artificially aged seeds.

### DA-6 treatment increases the concentration of soluble sugars in aged soybean seeds during imbibition

During seed germination and early seedling establishment, the energy supporting these biological processes comes primarily from carbon reserves stored in the seed itself, with sucrose and fructose being the main forms of these carbon reserves (Eastmond, 2004, 2006; Theodoulou and Eastmond, 2012). To investigate further the physiological and molecular mechanisms underlying the positive effects of DA-6 on germination and seedling establishment from aged soybean seeds, we next analyzed the concentrations of soluble sugars, namely sucrose and fructose, during imbibition of aged soybean seeds in the presence or absence of DA-6.

**Fig. 5.**
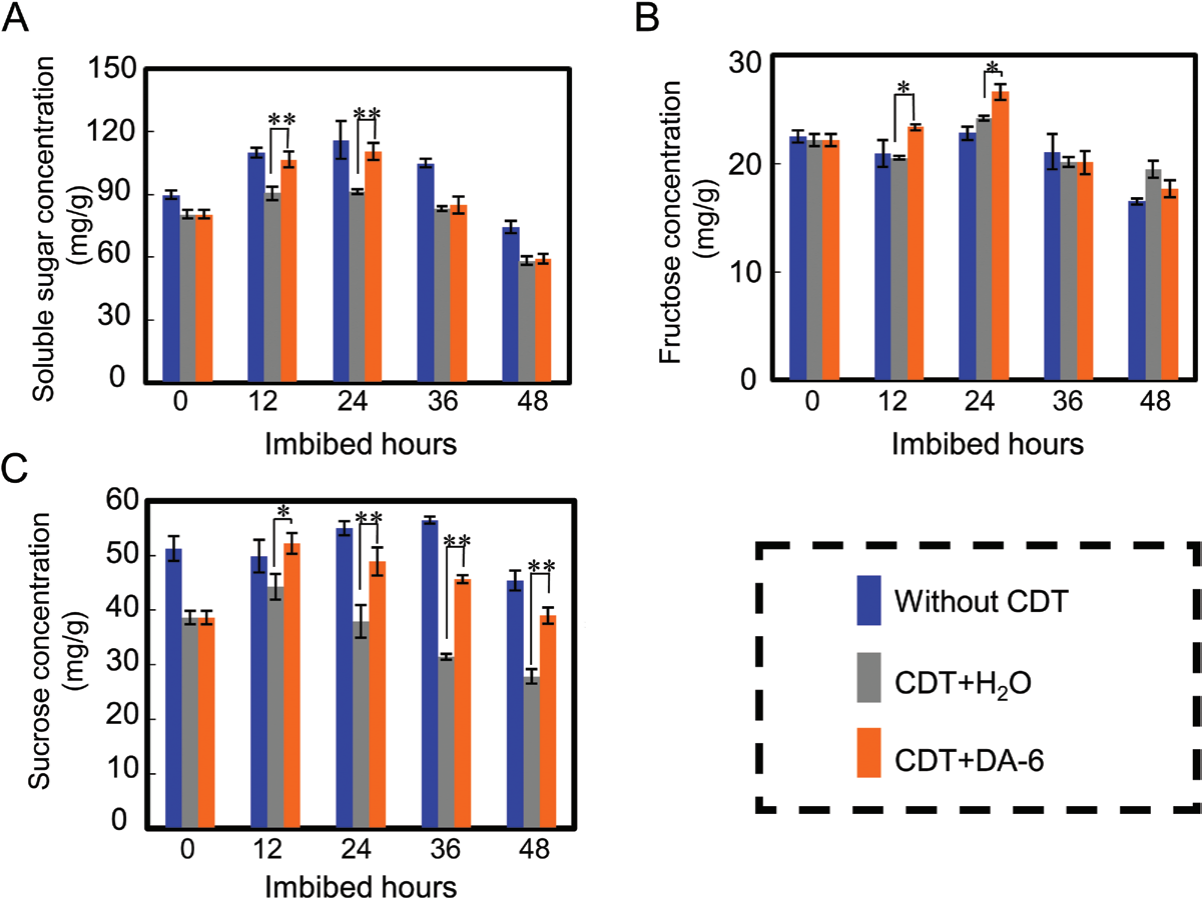
DA-6 treatment increases the concentration of soluble sugars during imbibition by aged soybean seeds. Different types of soybean seeds (healthy seeds without CDT, CDT seeds with H_2_O, and CDT seeds with DA-6 treatment) were employed. (A) Soluble sugar quantification analysis. (B) Fructose concentration quantification. (C) Sucrose concentration analysis. The average percentages of four repeats ±SE are shown. Asterisks (*) and (**) indicate a significant difference at *P*<0.05 and *P*<0.01, respectively, by Student’s *t*-test analysis. A 200 μM concentration of exogenous DA-6 was used.

During soybean seed imbibition, the concentration of total soluble sugars increased initially, before decreasing (Fig. 5A). The concentration of total soluble sugars in DA-6-treated CDT-aged soybean seeds was higher than that of aged seeds that had not been DA-6 treated, especially after 12 h and 24 h imbibition (Fig. 5A). The soluble sugars in seeds are predominantly sucrose and fructose, so we then investigated the effect of DA-6 on these two sugars. The results revealed that, after exogenous DA-6 treatment, the concentrations of fructose and sucrose in CDT-aged soybean seeds were higher than those in CDT-aged seeds without DA-6 treatment (Fig. 5B, C). It is noted that the CDT+DA-6 seeds showed a higher fructose content than both control and CDT-aged seeds at some time points (Fig. 5B). These results suggested that DA-6 promoted the germination and seedling establishment of aged soybean seeds by increasing the concentrations of soluble sugars, principally sucrose.

### DA-6 increases the concentrations of several fatty acids during imbibition of aged soybean seeds

It is well known that, during the germination of and early seedling establishment from seeds of oilseed crops, hydrolysis of triacylglycerol (oil) releases fatty acids and glycerol, which, in turn, are converted by gluconeogenesis to produce different types of soluble sugar (Eastmond, 2004, 2006; Quettier and Eastmond, 2009; Theodoulou and Eastmond, 2012). To better understand the causes of the increase in soluble sugar concentrations in CDT-aged soybean seeds after DA-6 treatment, we quantified the levels of several fatty acids during soybean seed imbibition.

**Fig. 6.**
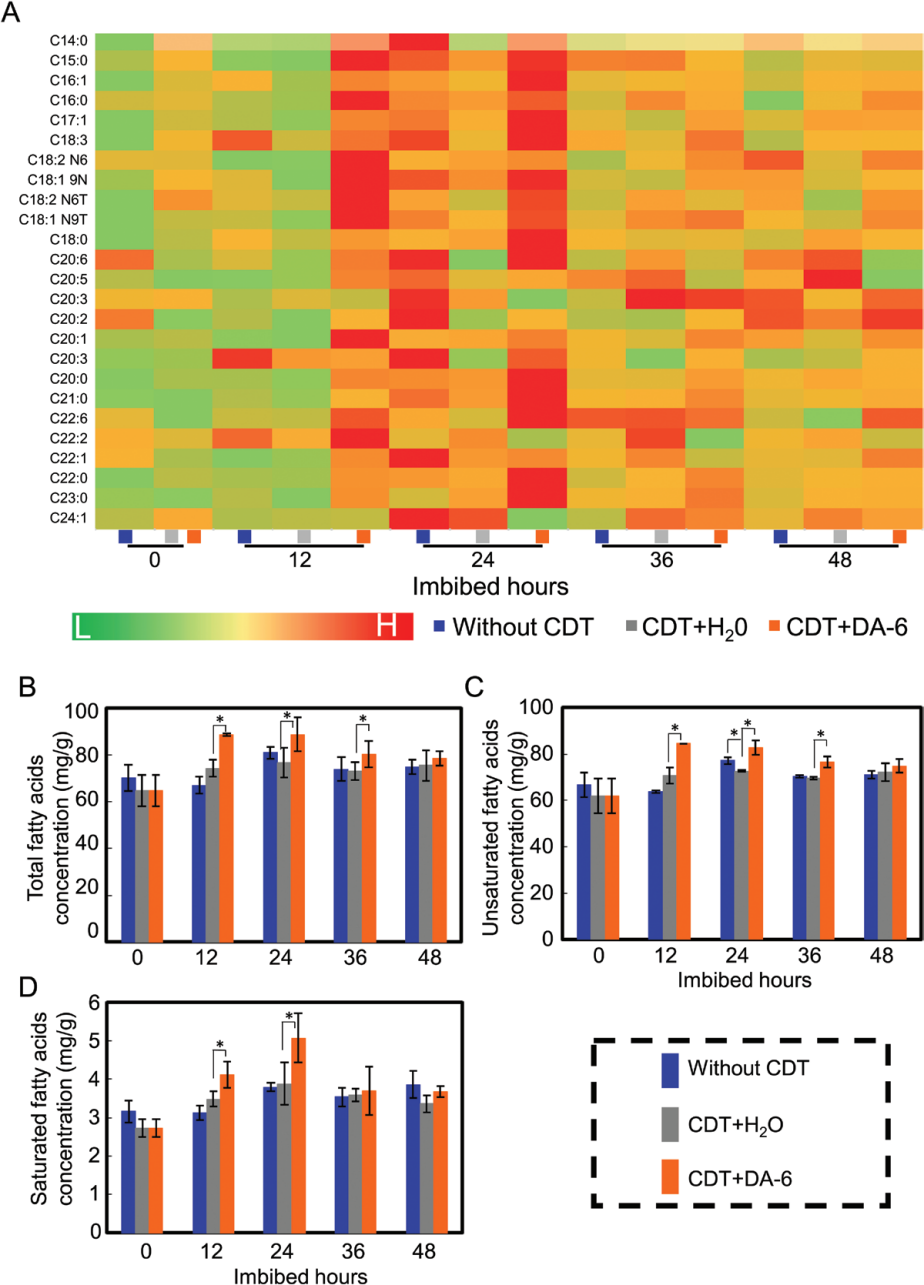
DA-6 treatment increases the concentration of total fatty acids in aged soybean seeds during imbibition. Different types of soybean seeds (healthy seeds without CDT, CDT seeds with H_2_O, and CDT seeds with DA-6 treatment) were employed. (A) The heat map analysis of the contents of several types of fatty acids during the soybean seed imbibition process with the time-course. The heat map was created by the Illustrator software. The fatty acid level from low (L) to high (H) indicates the minimum and maximum in the entire database. (B) The total fatty acid concentration in different types of soybean seeds during imbibition. (C) The unsaturated fatty acid concentration in soybean seeds during imbibition. (D) The saturated fatty acid concentration in soybean seeds during imbibition. The average percentages of four repeats ±SE are shown. Asterisks (*) and (**) indicate a significant difference at *P*<0.05 and *P*<0.01, respectively, by Student’s *t*-test analysis. A 200 μM concentration of exogenous DA-6 was employed.

GC-MS analysis showed that, after 24 h imbibition, the concentrations of most of the fatty acids in untreated soybean seeds increased (Fig. 6A). In seeds artificially aged by CDT, this increase in fatty acid concentrations after 24 h imbibition was largely absent (Fig. 6A). However, exogenous DA-6 application to CDT-aged seeds fully restored the fatty acid profile, with the concentrations of most of the fatty acids in DA-6-treated CDT seeds being higher than those in seeds without DA-6 treatment after both 12 h and 24 h imbibition (Fig. 6A). The concentrations of total, unsaturated, and saturated fatty acids in the DA-6-treated CDT-aged soybean seeds were all higher than the corresponding levels in CDT seeds in the absence of DA-6 treatment at most of the time points (Fig. 6B–D). It is noted that the level of unsaturated fatty acids in CDT+H_2_O seeds is significantly lower than that in seeds without CDT after 24 h imbibition (Fig. 6C). Similar to this, the level of total fatty acids in CDT+H_2_O seeds also decreased compared with the seeds without CDT, although the difference was not significant (Fig. 6B). Finally, we investigated the effects of DA-6 treatment on the concentration of each of the primary types of fatty acids in soybean seeds, namely palmitic, stearic, oleic, linoleic, arachidic, and linolenic acids. The results showed that DA-6 treatment increased the concentration of each fatty acid in aged soybean seeds during imbibition (Supplementary Fig. S5). Taken together, these findings indicated that DA-6 positively regulates the conversion of triacylglycerol to fatty acids, which are important precursors for the production of soluble sugars.

### DA-6 increases the transcription of several key genes involved in the conversion of triacylglycerol to fatty acids and sugars in aged soybean seeds during imbibition

**Fig. 7.**
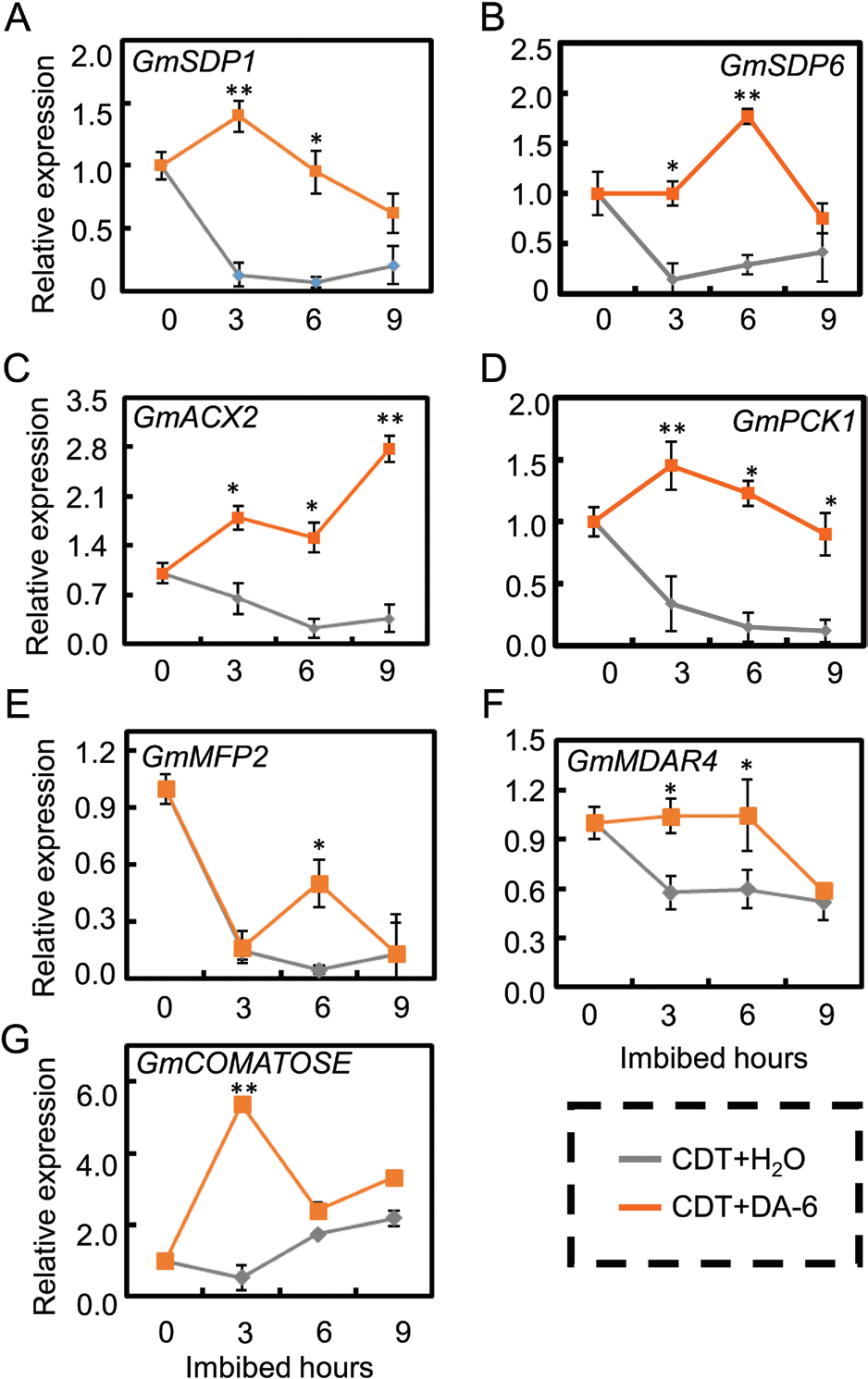
Positive effect of DA-6 on the transcription of several key genes which are involved in hydrolysis of triacylglycerol in aged soybean seeds during imbibition. Gene expression was investigated by qPCR assay during the course of the imbibition process. Different types of soybean seeds (CDT seeds with H_2_O and CDT seeds with DA-6 treatment) were employed. These different types of soybean seeds were employed for mRNA extraction, and three replications were performed. *GmSDP6* and *GmSDP1* encode FAD-G3P dehydrogenase and patatin-like domain-containing triacylglycerol lipase, and *GmACX2* and *GmPCK1* encode acyl-CoA oxidase and phosphoenolpyruvate carboxykinase, respectively. Those genes are all involved in the pathways by which the triacylglycerol was transferred to fatty acids and sugars during imbibition. (A) *GmSDP1*; (B) *GmSDP6*; (C) *GmACX2*; (D) *GmPCK1*; (E) *GmMFP2*; (F) *GmMDAR4*; (G) *GmCOMATOSE.* Asterisks (*) and (**) indicate a significant difference at *P*<0.05 and *P*<0.01, respectively, by Student’s *t*-test analysis. A 200 μM concentration of exogenous DA-6 was used.

We had observed that DA-6 treatment increased the concentrations of several fatty acids and soluble sugars in aged soybean seeds during imbibition (Figs 5, 6). Subsequently, we further analyzed the effects of DA-6 on increasing the conversion of triacylglycerol to fatty acids and sugars in aged soybean seeds during imbibition by studying the effects on the transcription pattern of key genes involved in the conversion of triacylglycerol to fatty acids and sugars. The results of qPCR analysis showed that the transcript levels of the key genes involved in the triacylglycerol hydrolysis pathway, namely *GmSDP1* (Fig. 7A), *GmSDP6* (Fig. 7B), *GmACX2* (Fig. 7C), *GmPCK1* (Fig. 7D), *GmMFP2* (Fig. 7E), *GmMDAR4* (Fig. 7F), and *GmCOMATOSE* (Fig. 7G), were all up-regulated (to varying degrees) in DA-6-treated aged soybean seeds during imbibition, compared with aged seeds without DA-6 application.

*GmSDP6* and *GmSDP1* encode FAD-G3P dehydrogenase and patatin-like domain-containing triacylglycerol lipase (Eastmond, 2004, 2006; Quettier *et al.*, 2008), respectively, which are the key enzymes in the hydrolysis of triacylglycerol. The expression levels of *GmSDP1* and *GmSDP6* in DA-6-treated aged soybean seeds were twice to three times those in seeds without DA-6 treatment (Fig. 7A, B). Similarly, the levels of *GmACX2* and *GmPCK1* transcripts detected in DA-6-treated aged soybean seeds were higher than in the corresponding aged seeds without DA-6 application (Fig. 7C, D). *GmACX2* and *GmPCK1* encode acyl-CoA oxidase and phosphoenolpyruvate carboxykinase, respectively, which are also involved in the process of β-oxidation of fatty acids (Li-Beisson *et al.*, 2010; Penfield *et al.*, 2012; Eastmond *et al.*, 2015). *GmPCK1* catalyzes the conversion of oxaloacetate (OAA) to phosphoenolpyruvate (PEP), the rate-limiting step in the metabolic pathway (Penfield *et al.*, 2012).

The accumulation of the transcripts of *GmMFP2*, *GmMDAR4*, and *GmCOMATOSE* after DA-6 treatment was also detected (Fig. 7E–G). These three genes are also involved in β-oxidation pathways (Rylott *et al.*, 2006; Eastmond, 2007; Kunz *et al.*, 2009). The accumulation of transcripts of these genes induced by DA-6 treatment might be expected to cause an increase in the production of soluble sugars. Altogether, the qPCR results suggested that DA-6 treatment of aged seeds increased the hydrolysis of triacylglycerol, as well as promoting the conversion of fatty acids to sugars during seed imbibition, by up-regulating expression of key genes involved in the respective pathways. The end-products of triacylglycerol hydrolysis, namely sucrose and fructose, provide the energy required to support the DA-6-induced germination and early seedling establishment from aged soybean seeds.

### DA-6 increases invertase activity in aged soybean seeds during imbibition

**Fig. 8.**
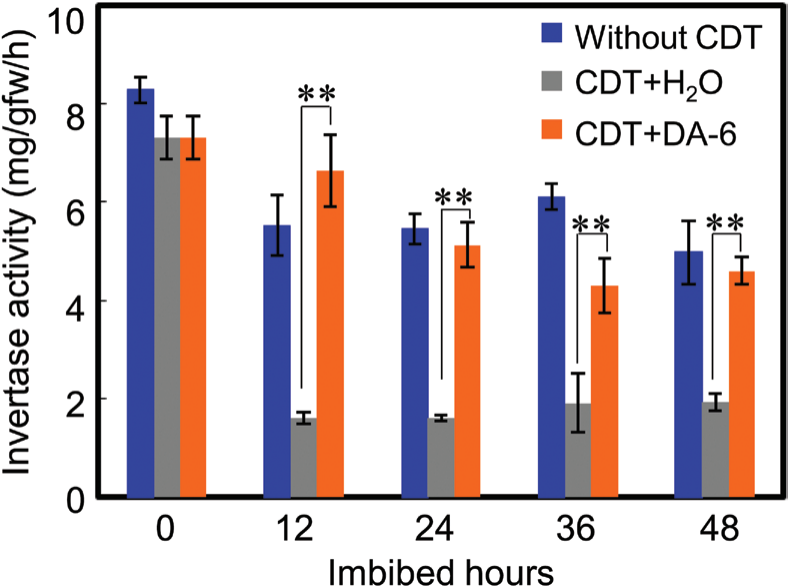
DA-6 treatment increases the invertase activity in aged soybean seeds during imbibition. Different types of soybean seeds (healthy seeds without CDT, CDT seeds with H_2_O, and CDT seeds with DA-6 treatment) were employed. The invertase enzyme activity in these distinct types of seeds was analyzed during imbibition. The average percentages of three repeats ±SE are shown. Asterisks (**) indicate a significant difference at *P*<0.01 by Student’s *t*-test analysis. A 200 μM concentration of exogenous DA-6 was used.

Invertase is the key enzyme catalyzing the hydrolysis of sucrose to give equimolar amounts of glucose and fructose (Jameson *et al.*, 2016; Wei *et al.*, 2016). We showed that, during germination of aged soybean seeds, DA-6 treatment increased the concentration of soluble sugars, including sucrose and fructose (Fig. 5). A previous study had demonstrated that suppressed invertase activity was associated with a delayed seed germination phenotype in Arabidopsis (Su *et al.*, 2016). To explore the relationship between DA-6 treatment, sucrose hydrolysis, and the germination ability of aged soybean seeds further, we quantified invertase activity during soybean seed germination. The results revealed that invertase activity was significantly repressed during imbibition of aged soybean seeds, compared with the activity in imbibed fresh seeds; while DA-6 treatment of aged seeds markedly increased invertase activity, compared with the seeds which had not been treated with DA-6 (Fig. 8). This finding was consistent with the positive effect of DA-6 on the germination and early seedling establishment of aged soybean seeds (Figs 2–4), and was in agreement with the increase in fructose concentration in DA-6-treated aged soybean seeds during imbibition (Fig. 5).

## Discussion

In the present study, phenotypic analysis, quantification of fatty acid and sugar concentrations, and analysis of gene expression and enzyme activity have demonstrated that the plant growth regulator DA-6 reverses the reduced germination and early seedling establishment rates exhibited by aged soybean seeds. The physiological and molecular mechanisms responsible for this positive effect are proposed to be as follows: in the aged soybean seeds, DA-6 promotes the hydrolysis of triacylglycerol to fatty acids and glycerol, and also enhances the conversion of fatty acids and glycerol to soluble sugars, which supply the energy needed during the soybean seed germination and early seedling establishment processes. This study also demonstrated that aged soybean seeds actually contain sufficient energy for germination, stored in the seeds as different food reserves, namely triacylglycerol, fatty acids, glycerol, and sugars. However, the aged seeds cannot use the storage energy, except for soluble sugars, because the conversion of triacylglycerol to soluble sugars appears to be blocked, resulting in the failure of seed germination and early seedling establishment.

### DA-6 has a positive effect on germination and early seedling establishment in aged soybean seeds

Numerous elegant studies have demonstrated that DA-6, an artificial plant growth regulator, has a range of biological functions which can impact on field crop production (Zhang *et al.*, 2008; Jiang *et al.*, 2012; He *et al.*, 2013; Salama *et al.*, 2014; Jiang *et al.*, 2015). The present investigation extended the practical uses of DA-6, demonstrating that DA-6 can reverse the loss of germination and seedling establishment activities associated with aging in soybean seeds.

Seed germination and subsequent seedling establishment are vital steps during the plant life cycle, contributing to the determination of plant distribution in wild species, and of yield in crops. Research from the laboratory, including the current study, has demonstrated that the decrease in seed germination ability as the seed ages is positively associated with the duration of the storage period (Yin *et al.*, 2014; Nguyen *et al.*, 2015; Yin *et al.*, 2015; Fleming *et al.*, 2017). In tillage agriculture, farm-saved seeds are stored by the farmer for varying periods of time, often under less than ideal conditions, resulting in seed aging and a decline in germination characteristics. Consequently, enhancement of the germination and seedling establishment from aged seeds is a worthwhile target in both basic and applied aspects of plant biology and crop production research.

Interestingly, a previous study had reported that hydrated graphene ribbon treatment could promote the germination of aged wheat seeds by increasing the concentrations of carbohydrate, amino acids, and fatty acids, and ensuring cell membrane integrity (Hu and Zhou, 2014). Ultrasonic treatment also improved seedling growth from aged grass seeds, including *Festuca arundinacea* Schreb. (tall fescue) and *Psathyrostachys juncea* L. (Russian wildrye), but the underlying mechanisms need further investigation (Liu *et al.*, 2016). A more recent study revealed that phytosynthesized silver nanoparticles increased the germination of aged rice seeds, with the mechanisms underlying this positive effect being suggested to include enhanced water uptake, rebooting of the reactive oxygen species (ROS) antioxidant systems in seeds, generation of hydroxyl radicals to achieve cell wall loosening, and nanocatalysis to accelerate starch hydrolysis (Mahakham *et al.*, 2017). However, most of these studies focused only on germination but not seedling establishment from aged seeds, and identified that there were diverse mechanisms underlying the effects promoting germination of aged seeds.

### DA-6 enhances the hydrolysis of triacylglycerol to sugars in aged soybean seeds

It is noted that the process of seed germination is distinct from that of seedling establishment, especially in aged soybean seeds. Germination analysis showed that percentage seedling establishment was generally lower than that of seed germination, especially in the soybean seeds stored for 10, 22, or 34 months (Fig. 1). For the soybean seeds stored for 34 months, the germination rate reached ~10%, but there was zero emergence (Fig. 1). These data suggested that the level of releasable energy stored in the seed itself is important for both processes (seed germination and seedling establishment); if the energy reserves were exhausted in the first stage (seed germination), then the germinated seed could not complete the second process (seedling establishment).

It is well known that the primary energy sources used by seeds during germination and seedling establishment are the soluble sugars, principally sucrose and fructose (Eastmond, 2004, 2006; Quettier *et al.*, 2008; Theodoulou and Eastmond, 2012). In oilseed species, including soybean and Arabidopsis, the hydrolysis of triacylglycerol produces fatty acids and glycerol, with the fatty acids and glycerol both being further converted into sucrose (Theodoulou and Eastmond, 2012). Consequently, these biochemical reactions, converting triacylglycerol to fatty acids and glycerol, and fatty acids and glycerol to sucrose, are vital for generating the energy to complete the seed germination and seedling establishment processes.

In the present investigation, we found that the germination ability of aged soybean seeds (following long-term storage) was lower than that of seeds stored for a shorter period (Fig. 1; Supplementary Fig. S1), and that DA-6 treatment significantly increased the germination and seedling establishment from aged soybean seeds (Figs 2–4; Supplementary Figs S2, S4). Subsequent research indicated that the conversion of seed storage oil (triacylglycerol) into sucrose during imbibition was insufficient to power germination of the aged soybean seeds in the absence of exogenous DA-6 application. Several lines of evidence supported this conclusion. DA-6 treatment markedly increased the levels of different types of fatty acids and soluble sugars (Figs 5, 6; Supplementary Fig. S5). In line with this, qPCR analysis further showed that DA-6 treatment increased the transcription of several key genes involved in the triacylglycerol hydrolysis pathway (Fig. 7).

**Fig. 9.**
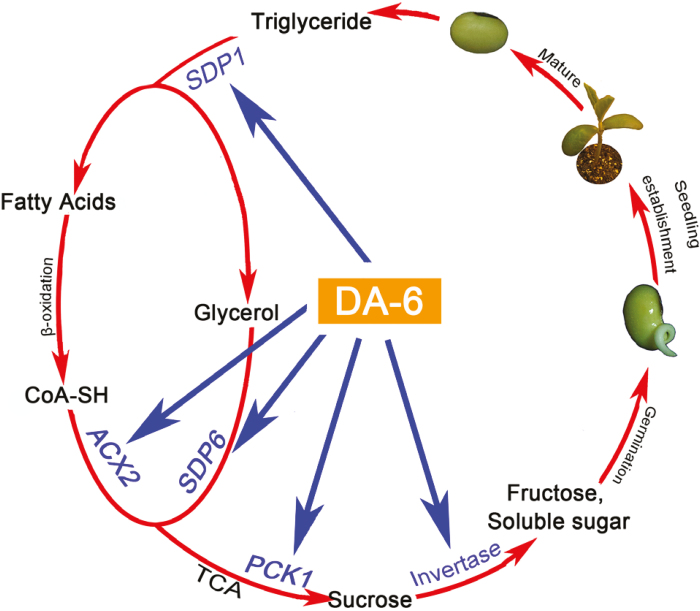
The proposed working model through which DA-6 promotes germination and seedling establishment from aged soybean seeds. In the aged soybean seeds, the transition from triacylglycerol to fatty acid and glycerol and then to the soluble sugars was blocked in the absence of DA-6. The application of exogenous DA-6 treatment in aged soybean seeds promoted the transcription of several key genes and elevated the activity of invertase which is involved in this pathway. Taken together, this model revealed that DA-6 promotes the germination and seedling establishment from aged soybean seeds by increasing the hydrolysis of triacylglycerol and the conversion of fatty acids and glycerol to sugars.

Taken together, the findings from the present investigation demonstrated that, during imbibition of aged soybean seeds, DA-6 increases the rate of hydrolysis of triacylglycerol to fatty acids and glycerol, and also the conversion of fatty acids and glycerol to sucrose and fructose by promoting the transcription of the key genes and the activity of the enzymes involved in those pathways (Fig. 9). On one hand, this study extended the range of agronomic and physiological crop processes identified as being affected by DA-6. On the other hand, the present investigation also demonstrated that the conversion of triacylglycerol to soluble sugars is important for the germination of aged seeds of oilseed crops.

Given the importance of the phytohormones abscisic acid (ABA) and gibberellins (GAs) in the regulation of seed germination (Shu *et al.*, 2013, 2015, 2016*c*, 2018), the relationship between DA-6 and the biosynthesis and signaling pathways of ABA and GA during germination of aged soybean seeds needs further investigation. For instance, does DA-6 regulate the biosynthesis and/or signal transduction pathways of ABA and GA? DA-6 is an artificial plant hormone, so the dissection of its receptor(s) will help us better understand the action of DA-6. Furthermore, the relationship between the DA-6 receptor(s) and ABA and GA also needs further exploration. Future studies should address whether DA-6 is also involved in the regulation of germination and early seedling establishment in aged seeds of the cereal crops (such as wheat, rice, and maize).

## Supplementary data

Supplementary data are available at *JXB* online.

Fig. S1. Natural aging significantly decreases soybean seed germination ability.

Fig. S2. DA-6 promotes germination ability of natural aged soybean seeds

Fig. S3. DA-6 has no effect on germination and seedling establishment of fresh soybean seeds.

Fig. S4. DA-6 treatment increases the total chlorophyll content and dry weight in seedlings which germinated from CDT-aged soybean seeds.

Fig. S5. DA-6 treatment increases the concentration of several types of fatty acids in aged soybean seeds during imbibition.

Table S1. Primer sequences used in this study.

Supplementary Figures S1-S5 and Table S1
