# Supplementary material for: DA-6 promotes germination and seedling establishment from aged soybean seeds by mediating fatty acid metabolism and glycometabolism
Source: J Exp Bot. 2018 Jul 3;70(1):101–14. doi: 10.1093/jxb/ery247 (PMC6305204; doi:10.1093/jxb/ery247)
Supplement: Supplementary Figures S1-S5 and Table S1 [file ery247_suppl_supplementary_figures_s1-s5_table_s1.pdf]

Zhou et al., Fig. S1

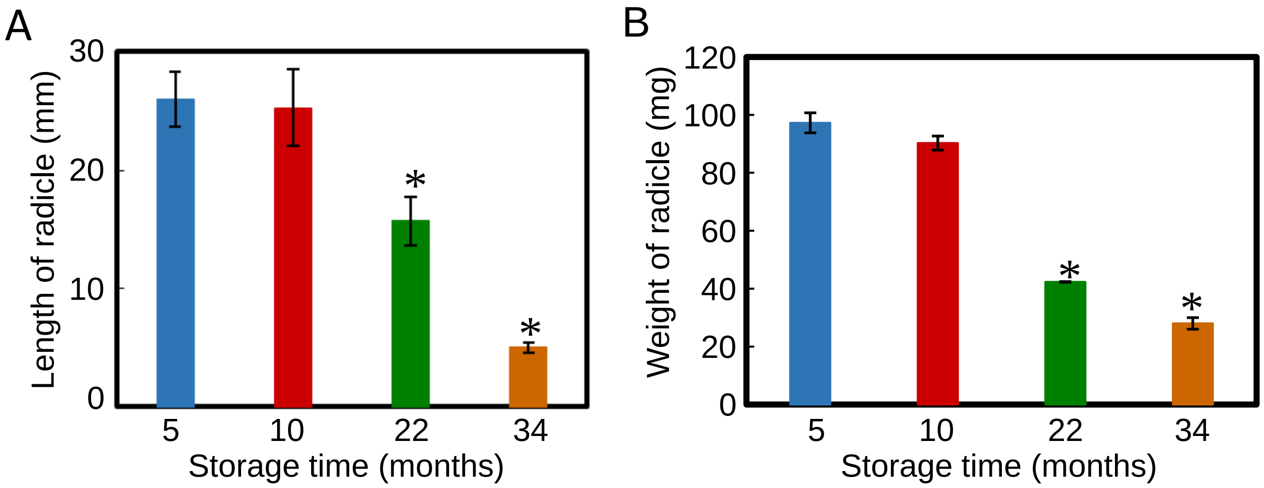

Supplemental Figure S1. Natural aging significantly decreases soybean seed germination ability.

(A) The radical length of germinated soybean seeds were measured (48 hours after sowing). (B) The fresh weight of germinated soybean seeds were quantified (48 hours after sowing). The soybean seeds were stored for 5, 10, 22 or 34 months after harvest and subjected to analysis. The average percentages of three repeats ± standard error are shown. The germination experiment were performed under 25 ° C and 60% relative humidity conditions. The asterisk (*) indicates a significant difference at the *P<0.05* level by Student's *t*-test analysis.

Zhou et al., Fig. S2

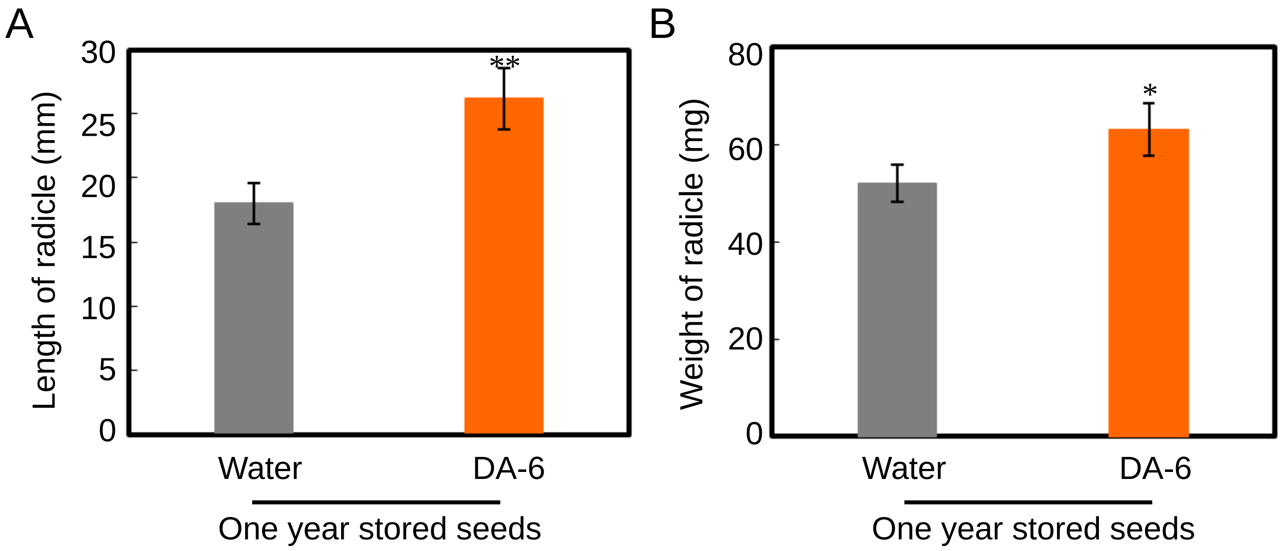

Supplemental Figure S2. DA-6 promotes germination ability of natural aged soybean seeds.

(A) The radical length of germinated soybean aged seeds were measured (48 hours after sowing), in the absence or presence of DA-6 treatment. (B) The fresh weight of germinated soybean aged seeds were quantified (48 hours after sowing), with or without DA-6 application. The soybean seeds were stored for 12 months after harvest and then subjected to analysis. The average percentages of three repeats ± standard error are shown. The germination experiment were performed under 25 ° C and 60% relative humidity conditions. The asterisk (*) indicates a significant difference at the $P<0.05$ level by Student's *t*-test analysis. 200 μM exogenous DA-6 was employed.

Zhou et al., Fig. S3

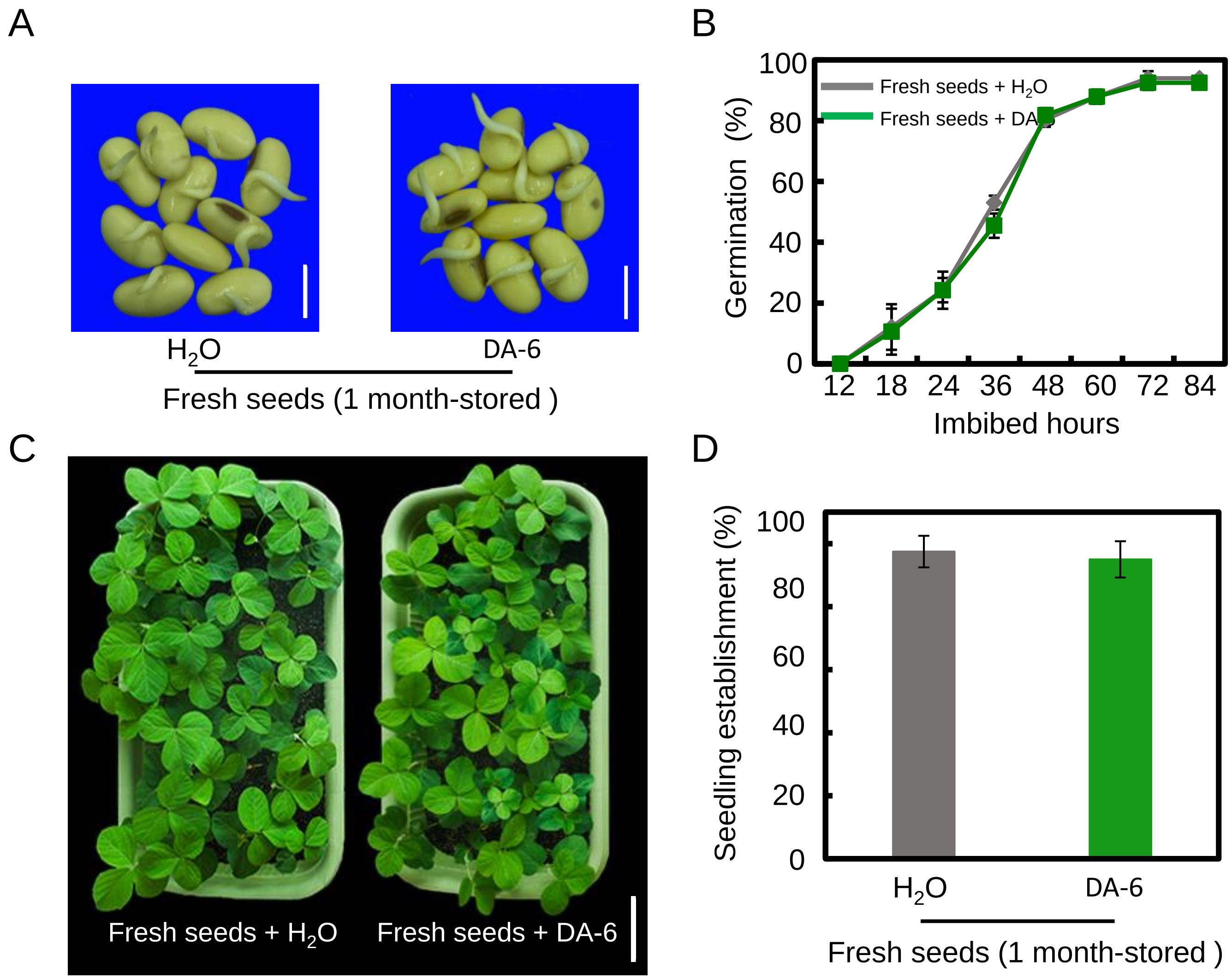

Supplemental Figure S3. DA-6 have no effect on germination and seedling establishment of the fresh soybean seeds.
(A) Representative photographs of fresh soybean seeds (stored for 1 month) during imbibition process (60 hours after sowing), with or without DA-6 treatment. Bar=10 mm. (B) The quantitative analysis of final germination rates of 1 month storage seeds in the absence or presence of DA-6 treatment are shown. (C) The early seedling establishment phenotype of 1 month storage seeds with or without DA-6 application. Bar =100 mm.

Zhou et al., Fig. S4

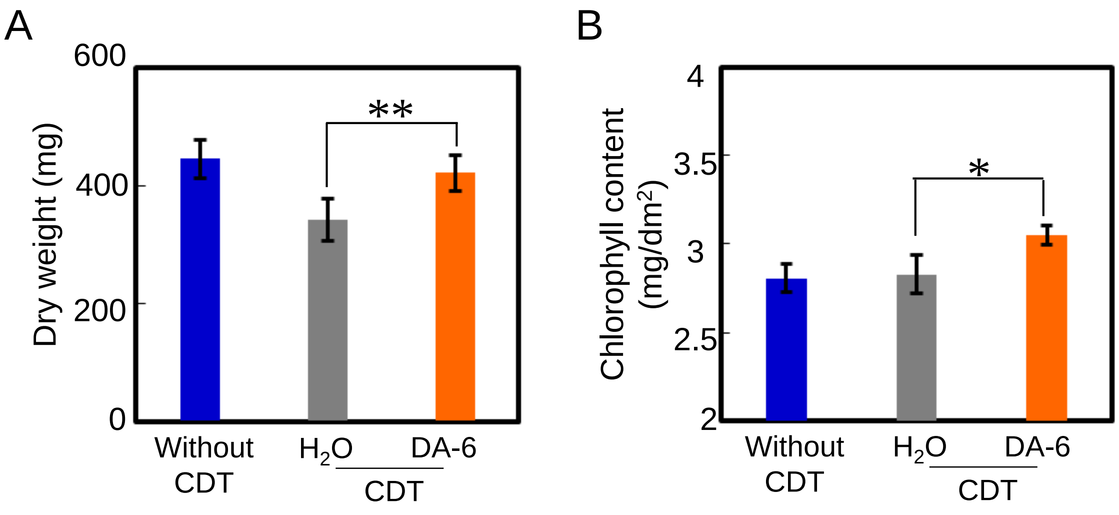

Supplemental Figure S4. DA-6 treatment increases the total chlorophyll content and dry weight in seedlings which germinated from CDT-aged soybean seeds.

(A) The dry weight of seedlings were measured with different types of soybean seeds (including the health seeds without CDT, CDT seeds with H_2O, and CDT seeds with DA-6 treatment). (B) The total chlorophyll content of seedlings were quantified with different types of soybean seeds. Percentages are the average of three repeats ± standard error. The seedling grown under 25 ° C and 16h light with 8h dark conditions. The asterisk (*) indicates a significant difference at the *P<0.05* level by Student's *t*-test analysis. 200 μM exogenous DA-6 was used.

Zhou et al., Fig. S5

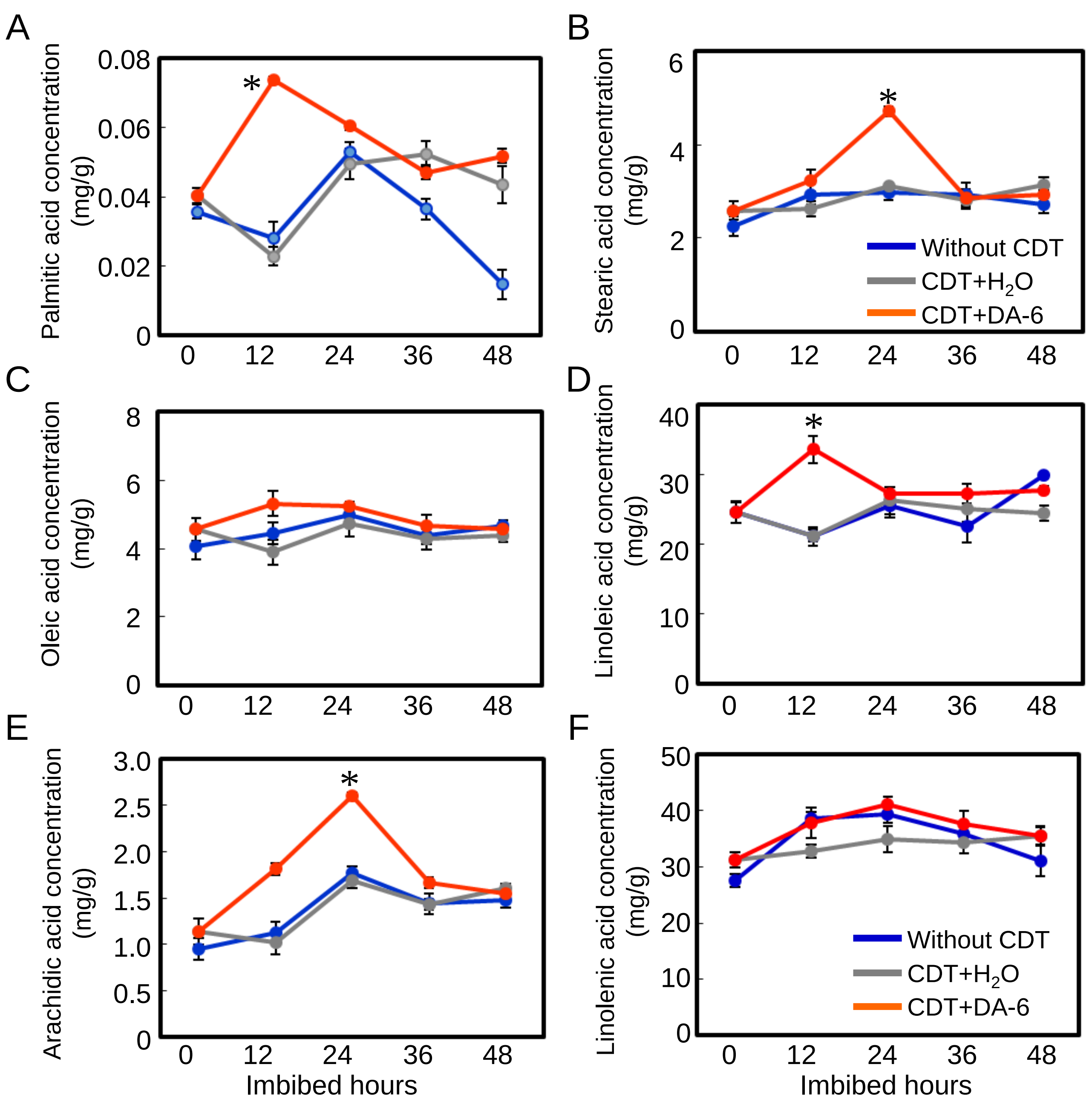

Supplemental Figure S5. **DA-6 treatment increase several types of fatty acids concentration in aged soybean seeds during imbibition.**
Different types of soybean seeds (including the health seeds without CDT, CDT seeds with H_2O, and CDT seeds with DA-6 treatment) were employed, during seed imbibition process. (A) Palmitic acid concentration. (B) Stearic acid level. (C) Oleic acid concentration. (D) Linoleic acid level. (E) Arachidic acid concentration. (F) Linolenic acid level. The average percentages of three repeats ± standard error are shown. The asterisks (*) and (**) indicate the significant difference at the P<0.05 and *P<0.01* level, respectively, by student's *t*-test analysis. 200 μM exogenous DA-6 was employed.

Supplemental Table 1. Primers for the qPCR in this study.

Gene	Forward primer	Reverse primer
GmTubulin	AACCTCCTCCTCATCGTACT	GACAGCATCAGCCATGTTCA
GmSDP1	TTCAAGGCACACAACACCTG	CATCCACCTCCTTCCCTTCAG
GmSDP6	CCAAGAAGGGAGTGACCTTCC	ACTGACCGTAGCAGCAATCC
GmPCK1	ACCTAAGTTACATGGTGCAGTG	CGTGCTCTGTTCCGTTCCTT
GmACX2	TGCTCTGAAGCTCGTCTGTG	CGAGCTTGTGGATTGCCTTG
GmMFP2	GCTGCTGAGGTGAGTGAGTAG	GTATCACGGCCCGTTGGAA
GmCOMATOSE	CACCTTTCAATAGGTTGATGTTCCA	GCAGTTCCACCAGCGATTAAG
GmMDAR4	TAGCCAGAAACCGGTGGTG	GCAATAATAACCCCGGCTGTG
